# Laryngopyocele: A Rare Cause of Upper Airway Compromise

**DOI:** 10.7759/cureus.89390

**Published:** 2025-08-05

**Authors:** Gulistan Bano, KSBS Krishna Sasanka, Swaha Panda, Tejaswi Mishra, Bhartendu Bharti, Saurabh Varshney, Pradosh Kumar Sarangi

**Affiliations:** 1 Otolaryngology - Head and Neck Surgery, All India Institute of Medical Sciences, Deoghar, Deoghar, IND; 2 Radiodiagnosis, All India Institute of Medical Sciences, Deoghar, Deoghar, IND

**Keywords:** airway obstruction, external cervical approach, hoarseness, laryngopyocele, tracheostomy

## Abstract

Laryngocele is defined as the abnormal dilatation of the laryngeal saccule by air, and when it becomes infected, it is termed a laryngopyocele. Laryngopyoceles can present acutely with airway compromise and swallowing difficulties, along with other symptoms such as hoarseness and neck pain. A 78-year-old male with a history of chronic obstructive pulmonary disease presented with a progressively enlarging left-sided neck swelling over 30 years, recently associated with hoarseness, dysphagia, and respiratory distress. Examination and imaging confirmed a mixed laryngopyocele causing significant airway compromise. An emergency tracheostomy was performed, followed by definitive excision via an external cervical approach. Intraoperatively, 12 mL of pus was drained, and histopathology revealed an inflamed cyst lined by respiratory epithelium. The patient recovered well, with successful decannulation and complete resolution of symptoms. Mixed laryngopyoceles are uncommon but may present as airway emergencies, and early recognition, prompt airway management, and complete surgical excision are essential for optimal outcomes.

## Introduction

Laryngocele is the herniation of the saccule of the laryngeal ventricle when it is filled with air and communicates with the larynx's lumen. Laryngoceles have been classified into three types based on whether they extend outside through the thyrohyoid membrane, internally obscure the airway, or have both components - external, internal, and combined or mixed laryngoceles. Laryngomucocele is formed when there is compression of the neck of the laryngocele, leading to hypersecretory action of the mucus glands. The stasis and infection of the secretion with subsequent time leads to the formation of laryngopyocele, and the patient can present with signs and symptoms of acute respiratory comprise, dysphagia, fever, and painful increase in the size of the swelling. The incidence of laryngopyocele in laryngoceles is around 5%-8% [1]. Here, we report a case of a mixed laryngopyocele that resulted in airway obstruction and was managed through an initial emergency tracheostomy followed by excision via the external cervical approach.

## Case presentation

A 78-year-old male presented to the outpatient department of a tertiary care center in Eastern India with a primary complaint of a persistent swelling on the left side of the neck, present for the past 30 years. Over the preceding three months, he reported progressive symptoms, including pain over the swelling, dysphonia, dysphagia, and dyspnea. The respiratory difficulty was episodic and characterized by intermittent stridor, which worsened in the supine position, leading the patient to preferentially adopt a lateral decubitus posture. He had a known history of chronic obstructive pulmonary disease and was a priest by occupation.

**Figure 1:**
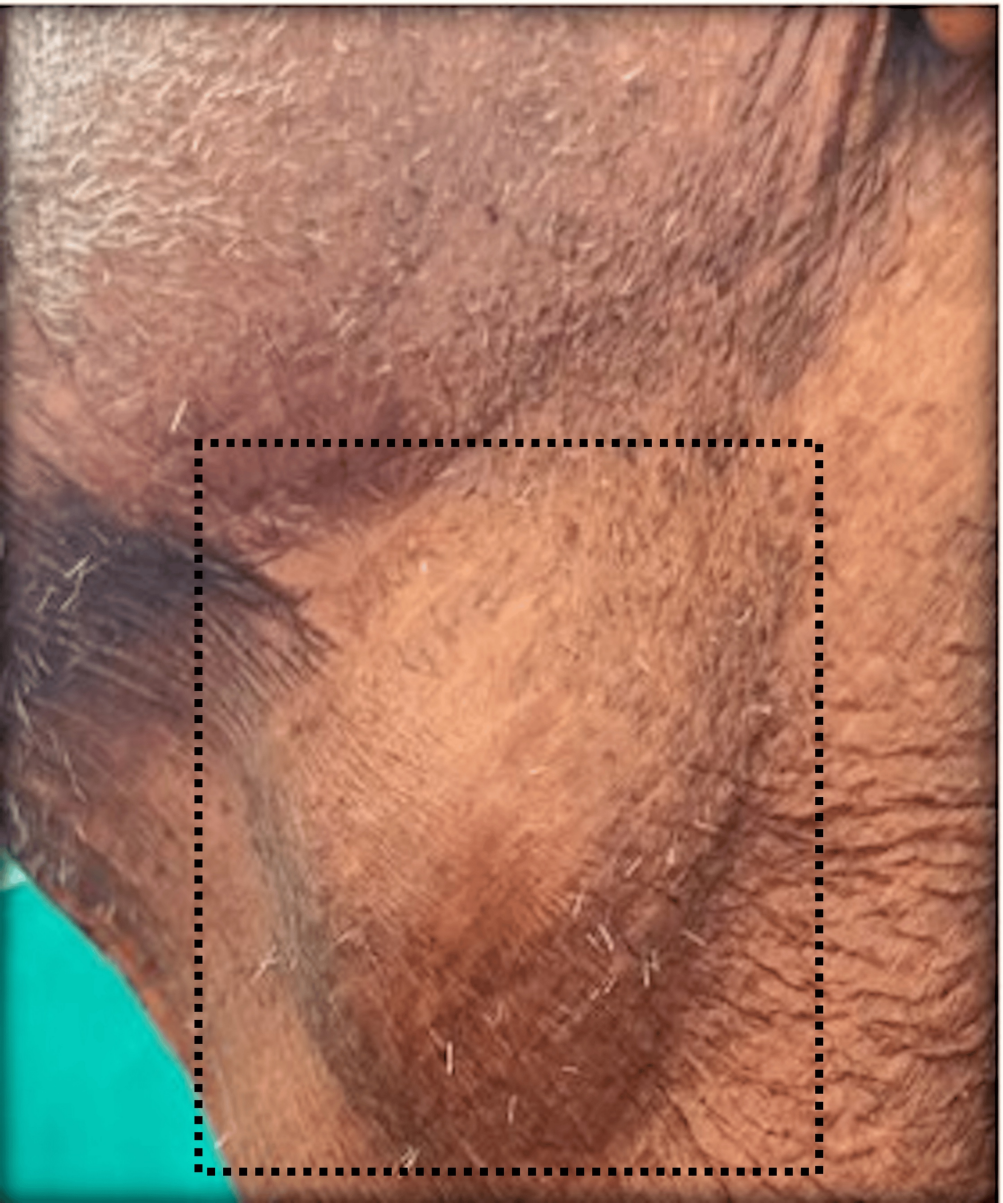
Clinical photograph showing a 3 × 3 cm swelling in the left upper neck, located below the angle of the mandible and anterior to the sternocleidomastoid muscle.

On clinical examination, a spherical swelling measuring approximately 3 × 3 cm was observed in the left upper cervical region (Figure 1). The swelling was soft in consistency, with mild tenderness and localized warmth. Notably, its size increased with coughing. The patient presented with tachycardia (pulse rate: 110/min) and experienced respiratory distress even at rest.

**Figure 2:**
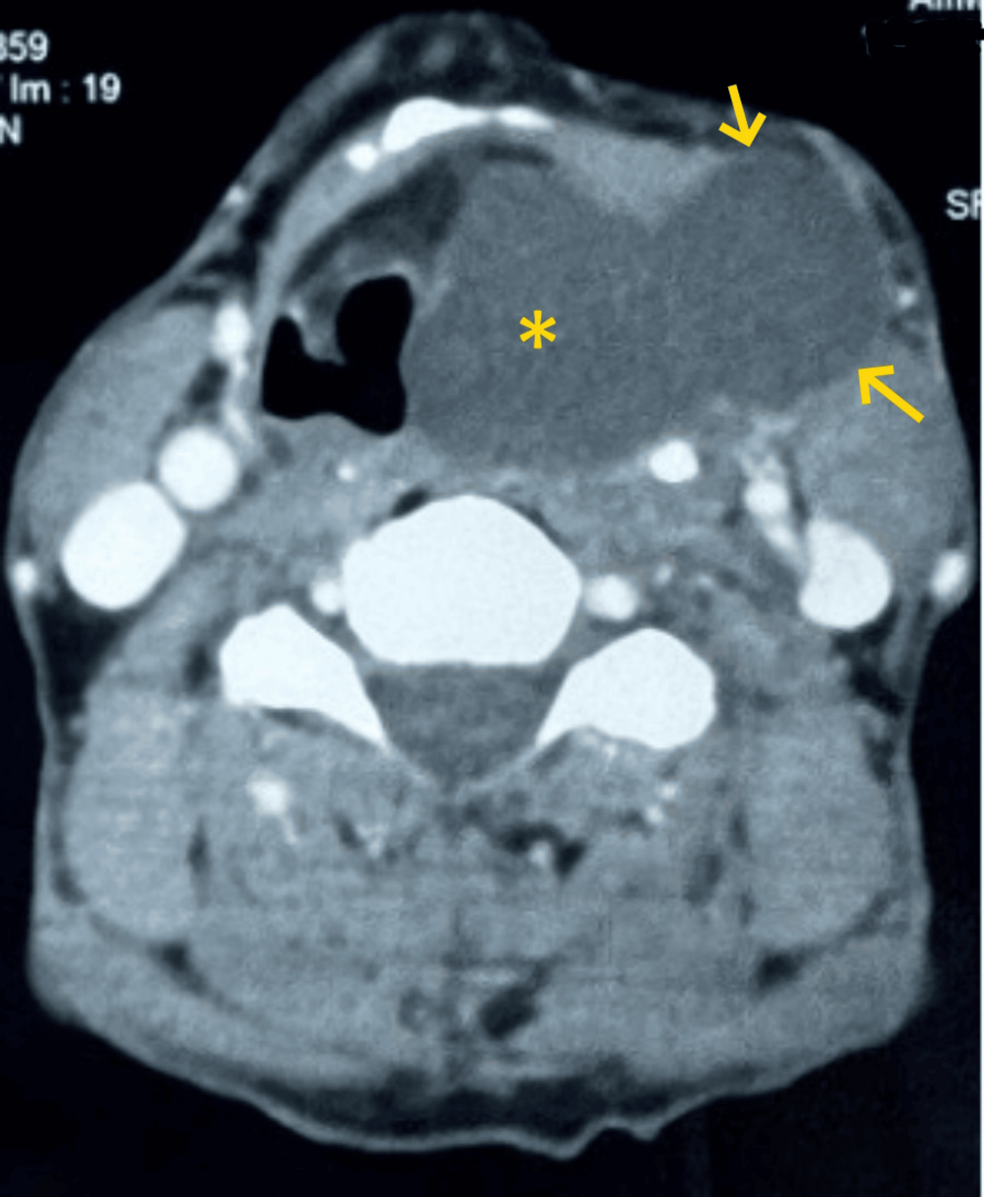
Contrast-enhanced computed tomography (CECT) of the neck showing a cystic lesion in the left level III cervical region, extending from the left anterior visceral space (*) into the left anterior cervical space (arrows). The lesion displaces the epiglottis and the left aryepiglottic fold toward the contralateral side.

Videolaryngoscopic evaluation demonstrated a smooth, bulging mass in the left lateral pharyngeal wall, extending to involve the epiglottis, aryepiglottic fold, and false vocal cord, leading to narrowing of the glottic chink. Ultrasonography revealed a cystic lesion containing internal debris, measuring approximately 44 × 23 mm, located in the left upper cervical region. Contrast-enhanced computed tomography (CECT) of the neck identified a cystic lesion in the left level III cervical region, extending from the left anterior visceral space into the left anterior cervical space. This lesion displaced the epiglottis and the left aryepiglottic fold toward the contralateral side. Additionally, obliteration of the left pyriform sinus and left laryngeal ventricle was noted, with associated edema of the vocal cords, as shown in Figure 2. Fine-needle aspiration cytology findings were consistent with an abscess.

**Figure 3:**
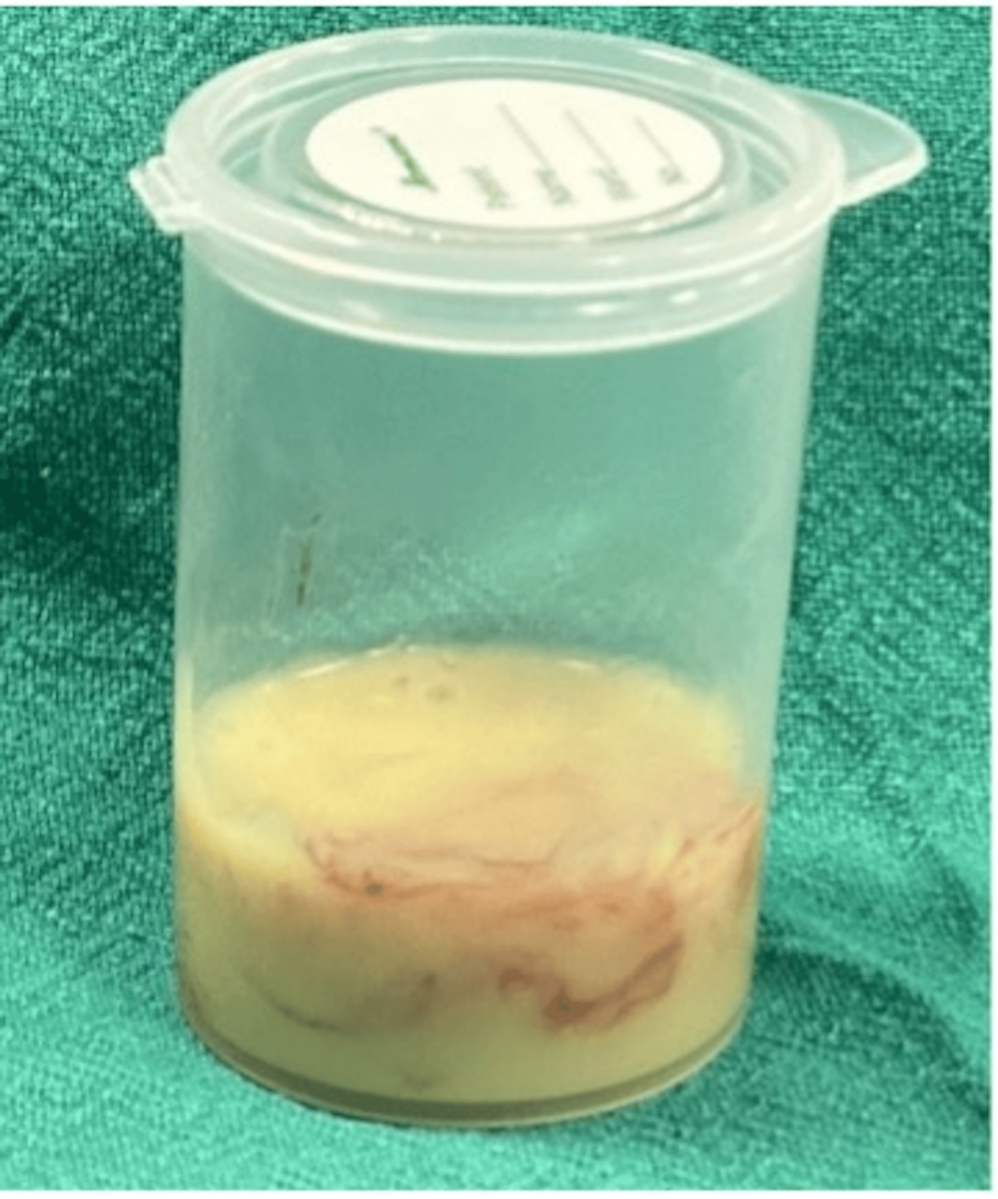
Specimen bottle containing approximately 12 mL of pus drained from the laryngopyocele.

**Figure 4:**
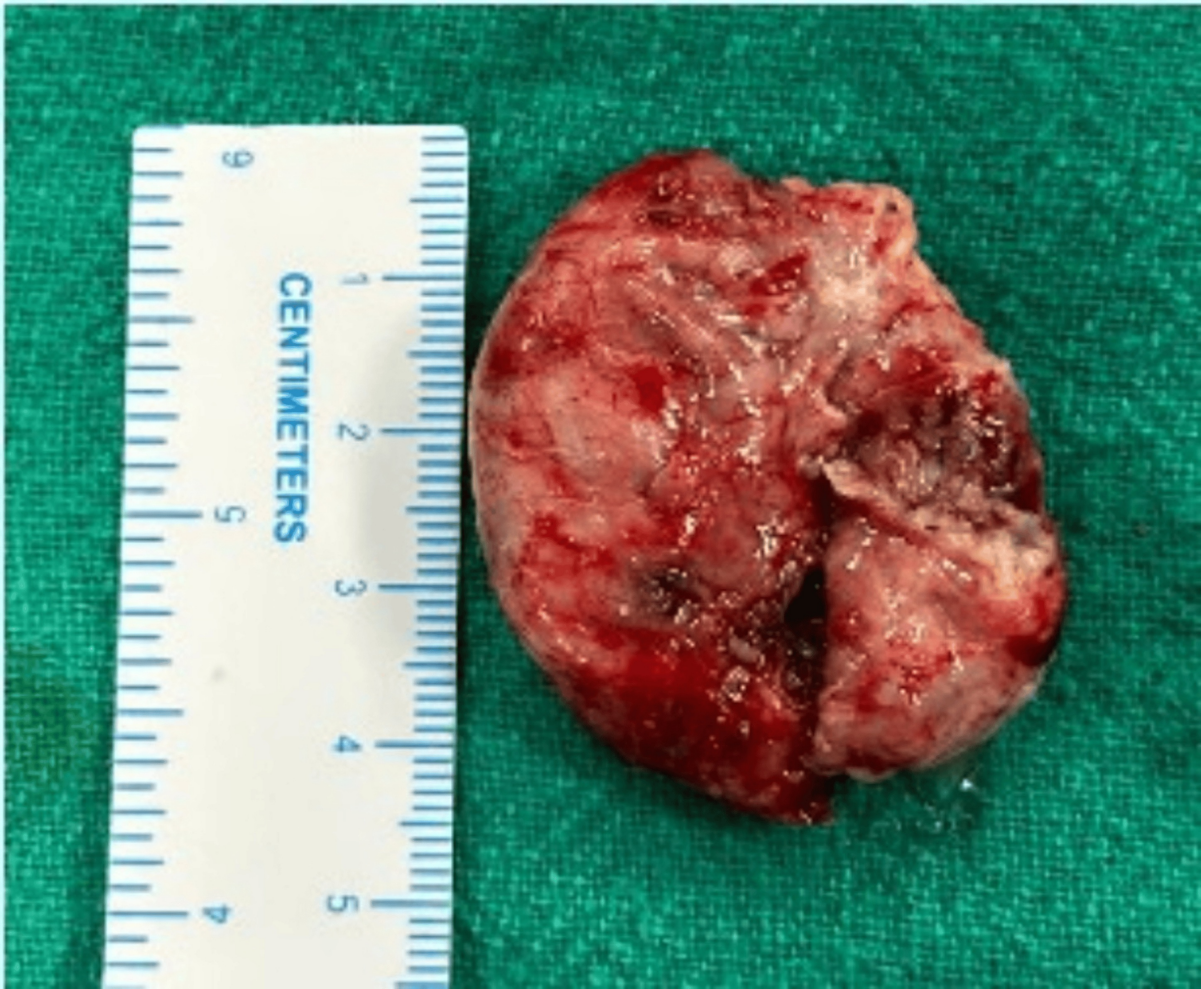
Gross specimen of the surgically excised laryngocele.

Due to significant respiratory distress, the patient underwent an emergency tracheostomy. Empirical broad-spectrum intravenous antibiotics were initiated, including injection Augmentin (1.2 g IV every eight hours), injection amikacin (500 mg IV every 12 hours), and injection metronidazole (500 mg IV every eight hours), all administered for five days. The patient was scheduled for surgical excision of the laryngopyocele via an external transcervical approach under general anesthesia. A horizontal cervical incision was made at the level of the thyroid notch, followed by elevation of the subplatysmal flaps. The laryngopyocele sac was identified, and its external aspect was incised to facilitate drainage of the pus. The proximal end of the sac was then grasped and meticulously dissected circumferentially, tracking it through the thyrohyoid membrane up to the level of the laryngeal saccule. The lesion was found to be attached to the thyroid cartilage. Complete excision of the sac was performed, and the resulting defect in the thyrohyoid membrane and lateral pharyngeal wall was surgically repaired. Approximately 12 mL of purulent material was aspirated (Figure 3). Intraoperative pus was sent for culture and sensitivity, which showed no growth. The excised specimen was submitted for histopathological analysis (Figure 4).

Postoperatively, the patient was maintained on nasogastric feeding for 10 days. Stepwise decannulation of the tracheostomy tube was done. Follow-up over a one-month period demonstrated progressive resolution of edema in the aryepiglottic fold. Histopathological evaluation revealed cystic structures lined by respiratory-type epithelium, with areas of granulation tissue. The cyst wall exhibited a perivascular mixed inflammatory infiltrate.

## Discussion

A laryngocele is a rare, air-filled dilation of the laryngeal saccule (appendix of the laryngeal ventricle of Morgagni) that maintains communication with the laryngeal lumen. It exhibits a marked male predominance, occurring approximately seven times more frequently in males than in females, with peak incidence noted in individuals between the fifth and sixth decades of life. Around 75% of laryngoceles are unilateral, with no consistent predilection for either side [2]. Clinically, laryngoceles commonly present with dysphonia and a palpable cervical mass, which becomes more apparent during activities that elevate intrathoracic pressure, such as the Valsalva maneuver [2].

Laryngopyocele is a rare complication of the laryngoceles, where retained mucus secretions are infected. They can present with dysphagia, hoarseness, neck swellings, septicemia, and respiratory distress [3]. Laryngoceles are sometimes seen in people who play wind instruments, like trumpet players, or in glassblowers, due to prolonged increased pressure in the larynx. Tumors in the larynx, especially supraglottic carcinomas, can also block airflow and elevate intralaryngeal pressure, potentially leading to laryngocele formation. This blockage can act like a valve, allowing air to enter but not escape [4]. Systemic diseases such as scleroderma and systemic lupus erythematosus may also contribute to its development [5].

Computed tomography of the neck is highly effective for diagnosing laryngoceles by providing cross-sectional views, while magnetic resonance imaging can more accurately define the extent of the lesion and its relationship to the thyrohyoid membrane. Diagnosis typically relies on a combination of radiological imaging and endoscopic evaluation. Some cases, like the one reported by Mallik et al., have been mistaken for other neck masses, such as infected branchial cysts [6]. Other possible mimics include infected saccular cysts, cervical lymphadenitis, and extralaryngeal spread of head and neck cancers.

Laryngopyoceles can pose a serious risk to life, with four deaths reported in the literature [7]. Fatalities are usually due to airway obstruction from the enlarged pyocele or aspiration of pus into the lungs.

Treatment typically involves surgical excision, which may be performed through endoscopic, external, or combined approaches, depending on the laryngocele's type and size. A 2016 meta-analysis by Al-Yahya et al. found that the external approach is commonly used for mixed laryngopyocele, and endoscopes are used for internal ones. There was a reported recurrence of mixed laryngopyocele, which was managed previously by the endoscopic approach and then required revision surgery by external method [8]. The external approach provides superior visualization and workspace and reduces the risk of aspiration. With modern laser technology, complete excision is increasingly possible via endoscopy [5]. In the case presented here, the external cervical approach offered excellent exposure, and the patient recovered without complications.

## Conclusions

Mixed laryngopyoceles are rare but can present with acute airway obstruction. Early recognition and timely surgical intervention, including airway management and complete excision, are crucial for optimal patient outcomes. This case underscores the importance of a multidisciplinary approach in managing laryngopyoceles, particularly in high-risk patients with pre-existing comorbidities.
